# Gamification for Machine Learning in Surgical Patient Engagement

**DOI:** 10.3389/fsurg.2022.896351

**Published:** 2022-04-22

**Authors:** Jeremy A. Balch, Philip A. Efron, Azra Bihorac, Tyler J. Loftus

**Affiliations:** ^1^ Department of Surgery, University of Florida Health, Gainesville, FL, United States; ^2^ Department of Medicine, University of Florida Health, Gainesville, FL, United States; ^3^ Precision and Intelligent Systems in Medicine (PrismaP), University of Florida, Gainesville, FL, United States

**Keywords:** machine learning and AI, shared decision making, decision support, gamification, serious game

## Abstract

Patients and their surgeons face a complex and evolving set of choices in the process of shared decision making. The plan of care must be tailored to individual patient risk factors and values, though objective estimates of risk can be elusive, and these risk factors are often modifiable and can alter the plan of care. Machine learning can perform real-time predictions of outcomes, though these technologies are limited by usability and interpretability. Gamification, or the use of game elements in non-game contexts, may be able to incorporate machine learning technology to help patients optimize their pre-operative risks, reduce in-hospital complications, and hasten recovery. This article proposes a theoretical mobile application to help guide decision making and provide evidence-based, tangible goals for patients and surgeons with the goal of achieving the best possible operative outcome that aligns with patient values.

## Introduction

Interactions between patient, surgeon, and healthcare systems converge on a set of choices: when to cut, how to cut, or even if to cut, and how those choices intersect with patient values. These interactions are constantly evolving and must drive informed decision making between patients and surgeons. However, objective estimates of risk can be difficult to make. Machine learning can perform accurate, real-time assessments of surgical outcomes, but these technologies are often hindered by lack of usability and interpretability. Gamification may offer a solution. This article will examine available and potential applications that enhance patient buy-in, encourage pre-operative optimization, drive evidence-based decision making, and improve post-operative recovery.

Defined as the use of game elements in non-game contexts, gamification is primarily a tool of persuasion (1, 2). Elements can include points, rewards, badges, challenges, competitions, leaderboards, leveling-up, and avatars (3, 4). Similar concepts to gamification include serious games—a game whose primary objective is not for entertainment but for skill acquisition—and simulations—virtual representations of real-life events (5). The distinctions between these concepts are often blurred. The goal of gamification is to target individuals with limited motivation for change and—by providing extrinsic rewards—builds intrinsic motivation (3, 4). These strategies involve goal setting, feedback, reinforcement, and social connectivity (1, 3, 5).

Gamification was initially used in business to increase consumer engagement with advertisements, but the health care industry was quick to follow. There is now almost ubiquitous representation of gamification in weight loss, medication compliance, and fitness mobile applications (5, 6). However, few studies focus on the use of applications in the perioperative period. PubMed contains 49 articles using the search terms “gamification” and “surgery”, with focus largely on either surgical education or recovery from orthopedic procedures. Adding “machine learning” or “artificial intelligence” to the search terms yields no results. Nevertheless, there is interest in developing such tools. Michard reviewed potential uses of similar mobile applications in different perioperative settings, acknowledging that their full potential has yet to be reached (7). Similarly, Davaris et al. called for more digital games in improving the health literacy of surgical patients (8).

Research into conventional shared decision-making activities has revealed limited efficacy. A Cochrane review of forty studies showed that various combinations of personal and technological interventions may slightly improve mental health-related quality of life outcomes but had no difference in physical health-related quality of life outcomes or in regret to undergo an operation (9). These interventions were found to have both low retention and poor usability by both patients and providers. However, as health care decisions involve greater complexity, we need tools to help patients become “co-producers, co-designers, and co-producers” in their surgical plan (10).

**Figure 1:**
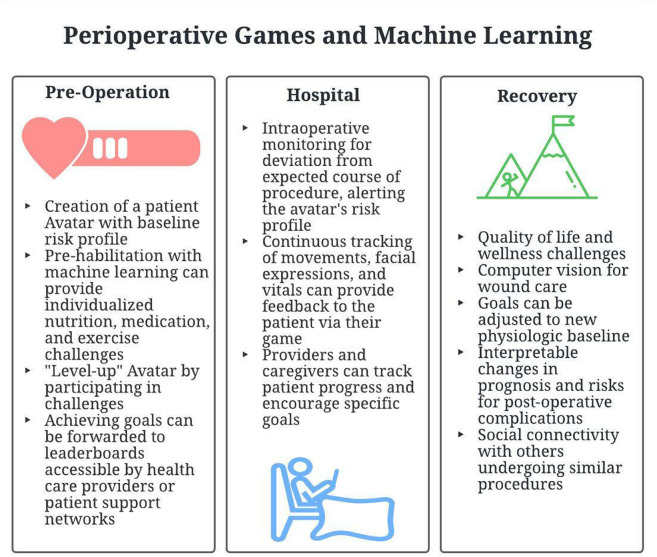
A summary of the potential application features.

In this paper, we will use a theoretical application to illustrate how machine learning can integrate with gamification for improved outcomes in elective operations. A summary of the potential application features are given in Figure 1

### The Patient Avatar

In elective surgery, the initial preoperative encounter between patient and surgeon is in a controlled setting. At that moment, the patient can be represented as a set of baseline variables of laboratory values, imaging, and medical and surgical history. In games, an avatar is a character that represents the player, in this case the patient. In our theoretical application, this avatar will initially be assigned fixed variables that can be used to predict operative outcomes and represent the start of the narrative as the patient and their avatar navigate through pre- and post-operative challenges.

Various prediction models already exist to estimate a patient’s baseline risk for post-operative outcomes. Linear prediction models are well known to most surgeons and include the American Society of Anesthesiologists Physical Classification System (ASA), the National Surgical Quality Improvement Program (NSQIP) Surgical Risk Calculator, and Surgical Outcome Risk Tool (SORT). These models have good accuracy for select postoperative complications and mortality (11). However, patient variables and outcomes are not independent, and the presence of multiple variables together are often greater than the sum of their individual contributions. Machine learning can account for this. Optimal Classification Trees are one such technology that allow for interpretable predictions. The Predictive Optimization Trees in Emergency Surgery Risk (POTTER) calculator outperforms ASA in predicting morbidity and mortality and is already available in application form (12). MySureryRisk can pull data from the Electronic Health Record, having evolved from a generalized additive model to a one using deep learning to provide real-time decision support (13, 14).

These techniques can be used to build an individualized avatar with assigned values to post-operative risks, such as wound complications, pulmonary issues, or cardiac events. A patient will then be able to directly see where they stand prior to a surgical intervention. This can be shown to a patient in clinic to start the conversation of operative risk and tailor decision-making to patient values and surgical options.

### Pre-Operative Training

Rare is the perfect operative candidate. At initial consultation, a surgeon will likely encourage their patients to reduce their risk factors by exercising, eating well, quitting smoking, or taking their medications.

A range of gamified health applications already exist to improve specific conditions with demonstrated efficacy. This has been improved with introduction of wearable technologies. In the BE FIT trial, Patel et al. randomized patients to wearable device that counted steps and gave feedback based on performance (15). A substantial number of patients in the intervention arm increased both the number of steps and percentage of days above step goal. Improved rates of smoking cessation and diabetes control have been demonstrated with the use of wearables (15, 16). Similarly, artificial intelligence-guided nutrition recommendations are being rapidly integrated into smart phone applications (17).

Data uploaded from wearables, or manually entered into smart phones, can be analyzed using machine learning techniques. These can be used to upgrade the patient’s avatar, or “level up” prior to surgery. Various challenges can be placed, which include step goals, medication adherence, weight loss targets, or incentive spirometry volumes. Post-operative risks can be visibly downgraded, providing positive feedback to the user. These goals can be forwarded to the surgeon or family members, providing social support.

### Hospital Course

Any operation is an injury inflicted onto a patient, decreasing physiologic reserve. From a game perspective, the patient’s avatar will have sustained a decrease in function from baseline and it is up to the patient and their care team to navigate them safely toward a full recovery.

From skin incision to leaving the hospital, our ability to monitor patients has increased exponentially over the last decade. First, machine learning is rapidly integrating itself into the operating room itself. Subtle changes in vitals or laboratory values can suggest need for more intensive monitoring after surgery or alert surgeons and anesthesiologists to potential hazards (18). While still in development, the OR Black Box system (Surgical Safety Technologies, Toronto, Canada) is working towards capturing audiovisual and physiologic data to identify root-causes of complications and near miss events post-operatively (19, 20). Moreover, computer vision is now capable of tracking the motion of laparoscopic or robotic instruments and may one day note where surgeons have had to deviate from the standard procedure (20–22). These technologies may soon be available intra-operatively to identify risk for post-operative complications.

Similarly, in intensive care units and wards, we can continuously track not only vitals and labs, but also movements, facial expressions, and behaviors (23, 24). This allows for early identification of post-operatively complications such as bleeding, anastomotic leak, cardiac events, delirium, and failure to thrive (25, 26). This is particularly important in preventing failure to rescue—the inability to prevent a death secondary to a post-operative complication—and improving postoperative triage decisions (18, 27).

Our theoretical application could provide feedback to both the patient and the provider. Once a patient engages with their avatar, they will see the steps they can take to increase their odds of safe discharge. A mobile application could provide reminders to complete pulmonary exercises, walk a certain distance, or sleep during appropriate hours. At Cedar-Sinai hospital, wearable activity monitors were used to improve assessment of ambulation and were associated with length of stay after eight commonly performed operations (28). It could also track pain scores. Technology is already available to track facial expressions for pain and delirium and could work their way onto smart phone cameras (24).

These variables can be fed into their avatar to visualize associated changes in risks of post-operative complication and give patients a greater sense of agency in their immediate post-operative recovery and update clinicians on where they stand.

### Post-Discharge Care

Post-operative outcomes are largely tracked to the 30- or 90-day post-operative mark and long-term follow up is not necessarily the specialty of the surgeon. However, patients can often face chronic disability or changes in quality of life following their procedure. How can machine-learning guided gamification be used in this context? What does it mean to win the game?

Ideally, a patient will at least attain their pre-operative baseline. Gamified apps can continue to the track the fitness and medication compliance of their users but also track quality of life outcomes (29–32). Goals for improved physical, mental, social, and functional health can be set by the patient and physician. Progress towards these goals may be tracked remotely overtime. In a review of available telehealth technologies, including gamified applications, Berton et al. found that remote, virtual rehabilitation was not inferior to in-person visits for clinical outcomes in orthopedic patients (30). Smartphone accelerometer data has likewise already been studied in post-surgical recovery monitoring (33). Incremental progress towards recovery goals can be met with rewards, whereas failure to achieve these goals can prompt referrals to appropriate mental and physical therapists. Within the immediate post-operative window, wearable technologies can alert the patient of the need to return to the hospital.

Semi-supervised machine learning is being used in Physical Medicine and Rehabilitation science where data from accelerometers can be used to track gait, upper body motion, and falls and predict recovery or decline (34–36). These may track subtle movements or changes in physicality over time that may not be appreciated by a patient undergoing slow progress and may act as a motivating force. Wearable technologies and smart phone applications can also alert clinicians to risk of readmission. Kane et al. found that failure to achieve baseline step counts was associated with risk of readmission in colorectal surgery patients (37). A meta-analysis of studies in mobile health applications used by postoperative patients demonstrated reduced emergency department visits and hospital admissions (38). Meanwhile, deep learning has been trained to recognize skin lesions at a degree comparable to board-certified dermatologists (39). In surgery, applications for wound tracking are in various stages of development, and one application, mPOWEr, is already available for download (40, 41). Through pictures of their surgical wound, patients can alert themselves and clinicians to early identification of surgical site infections and wound complications.

Finally, some patients may have a new physiologic baseline. Several applications exist to assist with rehabilitation in this setting. SCI Hard is a serious game designed by Michigan Medicine for adolescents following spinal cord operations and injury (42). The platform is built around arm impairment and spasticity and teaches healthy behavioral strategies for these patients. Integration with EEG and MRI studies can also identify patterns of neuromuscular recovery (23, 34).

Having a mobile application with regular, postoperative patient engagement could aid in patient recovery and reduce readmissions.

### Surgery, Games, and the Metaverse

While perhaps decades away, the integration of healthcare into the notion of an extended reality is highly likely. The concept of the metaverse is modeled off of ideas in literature and the massive multiplayer online games currently enjoyed by millions of users (43, 44). As avatars in the virtual world become ever more accurate representations of us, so too does the possibly of charting the different paths these avatars can take when subjected to a physiologic insult. These “digital twins” will be essential for guiding both physician and patient decisions (45). In addition, telemonitoring of our patients, both in the hospital and outside, will become a regular part of healthcare, expanding access to care, and ideally optimizing brick and mortar resources. However, these concepts are still in their infancy and deserve both further imagination and dedicated study in the coming years.

### Limits of Gamification for Surgical Patients

There are several limitations to the use of gaming concepts in healthcare. First, the notion of using game-strategies in possibly life-threatening illness may risk trivializing the patient experience. Design strategies must create an appropriate format to avoid alienating the user. Similarly, in patients who are extremely debilitated, games may be demotivating when they see that, no matter their actions, risks for complications after surgery remain high. It will be important to calibrate points and rewards based on incremental improvements.

Gamification can often appeal to those who need it least. Fitness applications are generally used by those who are already healthy. These applications must be designed to be engaging and result in both perceived and tangible rewards. A meta-analysis by the Edwards et al. used application rating as a proxy for health benefits and were not able to identify a relationship between specific game strategies and rating, though the users gave high ratings overall (46). In a systemic review, Looyestyn et al. showed that 40% of gamified approaches failed to improve motivation and engagement (47). This may be a result of personal preferences. While some may be motivated by leaderboards, others will prefer simple point systems. Still more will not engage well with technology at all. Researchers are currently working on personalized games that can adjust the framework of the game based on user preferences and game-design principles (48). These too can be guided by semi-supervised machine learning strategies. An ideal application would adapt to the user as they succeed or fail to meet certain goals.

Finally, there are ethical considerations. There is a danger in constantly collecting data on our behaviors, which may be used to adjust insurance policies or patient billing. Operating room Black Box systems have clear medicolegal implications if they were made available outside of Morbidity and Mortality conferences (20). Many of these applications are poorly regulated. In the UK, the National Health Service (NHS) Health Apps Library was initially launched in 2013 to provide lists of trusted applications but faltered when these applications were found to be sharing health information with developers (49). Relaunched in 2017, it continued to have difficulties with assessing application safety, privacy, interoperability, and useability; it was again decommissioned in 2021 (50). Without extensive review and testing of these applications by surgeons and perioperative care providers, we may inadvertently cause harm.

Gamification is just one strategy in a larger armamentarium for improving patient-surgeon decision making and outcomes.

## Conclusions

Gamification may render the technologies of machine learning more transparent to patients and their providers. In our theoretical application, a patient is assigned an avatar with a baseline risk profile for a specific operation. This avatar may “level up” as it progresses, along with its human counterpart, through various challenges of improved exercise, diet, and medication compliance. The avatar, like the patient, will undergo a decline physiologic reserve following an operation. Through machine-learning enabled interpretation of vitals, laboratory values, telemetry, movements, and behaviors, the avatar’s risk profile for post-operative complications can be visibly changed through patient actions, giving tangible feedback. After discharge, prompts for rehabilitation exercises and wound monitoring may lead to appropriate use of readmission, early clinic visits, or continued observation. With integration into the electronic health record, these applications may alert patients and physicians of impending complications before the patient is fully aware of a problem. Gamification through mobile applications is a promising strategy to bring machine learning into routine clinical practice.
